# Weakening the IF2-fMet-tRNA Interaction Suppresses the Lethal Phenotype Caused by GTPase Inactivation

**DOI:** 10.3390/ijms222413238

**Published:** 2021-12-08

**Authors:** Jerneja Tomsic, Enrico Caserta, Cynthia L. Pon, Claudio O. Gualerzi

**Affiliations:** 1 Laboratory of Genetics, Department of Bioscience and Biotechnology, University of Camerino, 62032 Camerino, Italy; jerneja.tomsic@gmail.com (J.T.); enrico.caserta@gmail.com (E.C.); scholzcarl@yahoo.de (C.L.P.); 2 City of Hope Comprehensive Cancer Center, Duarte, CA 91010, USA

**Keywords:** bacterial translation initiation, initiation factor IF2, GTP hydrolysis, dominant lethal phenotype, lethality suppression

## Abstract

Substitution of the conserved Histidine 448 present in one of the three consensus elements characterizing the guanosine nucleotide binding domain (IF2 G2) of *Escherichia coli* translation initiation factor IF2 resulted in impaired ribosome-dependent GTPase activity which prevented IF2 dissociation from the ribosome, caused a severe protein synthesis inhibition, and yielded a dominant lethal phenotype. A reduced IF2 affinity for the ribosome was previously shown to suppress this lethality. Here, we demonstrate that also a reduced IF2 affinity for fMet-tRNA can suppress this dominant lethal phenotype and allows IF2 to support faithful translation in the complete absence of GTP hydrolysis. These results strengthen the premise that the conformational changes of ribosome, IF2, and fMet-tRNA occurring during the late stages of translation initiation are thermally driven and that the energy generated by IF2-dependent GTP hydrolysis is not required for successful translation initiation and that the dissociation of the interaction between IF2 C2 and the acceptor end of fMet-tRNA, which represents the last tie anchoring the factor to the ribosome before the formation of an elongation-competent 70S complex, is rate limiting for both the adjustment of fMet-tRNA in a productive P site and the IF2 release from the ribosome.

## 1. Introduction

Initiation factor IF2 is a multi-domain GTPase belonging to the subfamily of translational GTPases [1] containing a GTP/GDP/ppGpp binding domain (IF2G-2) comprising the same highly conserved elements implicated in guanosine nucleotide binding and hydrolysis found in other ribosomal GTPases such as EF-Tu, EF-G, and RF3 [2,3,4]. IF2 binds to both 30S and 50S ribosomal subunits and plays an essential function in all the initiation steps of translation. Under optimal physiological conditions, IF2 is complexed with GTP, binds to the 30S ribosomal subunit, and promotes the 30S initiation complex (30S IC) formation by recruiting the initiator fMet-tRNA to the ribosomes [5]. In the presence of GTP, IF2 also favors the association of the 30S IC with the 50S ribosomal subunit to generate a 70S initiation complex (70S IC) which will eventually become an elongation-competent 70S EC ready to enter the elongation phase of translation (for a review see [6,7]). After the hydrolysis of IF2-bound GTP, which occurs immediately upon subunits association, IF2-GDP-Pi remains initially ribosome-bound and only after a rather long time lapse, the Pi is dissociated and the 70S IC “matures” to become a 70S EC. The nature of the events occurring during the long (~200 ms) time lag, which follows GTP hydrolysis and precedes 70S EC formation, has been the object of numerous structural [3,4,8,9,10,11,12,13,14,15] and kinetic [5,6,16,17,18,19,20,21,22,23] investigations. The results of these studies demonstrate that before an “initiation dipeptide” (i.e., the peptide made between fMet and the amino acid corresponding to the second mRNA codon) is formed and IF2 is recycled off the ribosomes, a number of conformational changes of the ribosome and of its ligands (fMet-tRNA and IF2) must take place. Overall, there is universal consensus that the structure of IF2-GTP is substantially different from that of IF2-GDP [3,8,24] and that the presence of IF2-bound GTP, unlike that of IF2-bound GDP, favors the 30S IC formation [25] and the joining of the 50S subunit to the 30S IC which triggers the immediate hydrolysis of GTP [6,16,17,20]. However, a rather controversial issue concerns the role played by the IF2-dependent GTP hydrolysis and by the energy generated during this process. As described in more detail in the Discussion, during the transition from 30S IC to 70S IC, the ribosomal subunits rotate with respect to one another to yield a semi-rotated ribosome, whereas a reverse rotation occurs to yield a non-rotated structure during the subsequent maturation of the 70S IC into an elongation-competent 70S EC. These movements are accompanied by several conformational changes of the main ligands (i.e., IF2 and fMet-tRNA) which remain ribosome-bound after the dissociation of IF1 and IF3. Upon Pi release, IF2 undergoes a transition from its GTP conformation to the GDP conformation while the initiator tRNA is repositioned on the ribosome, going from an initial so-called P/pi (or P/I) position to a P/P position [8,9,10,14,15]. As to the role played by GTP hydrolysis, one point of view is that the energy generated by this chemical reaction drives the conformational changes of the factor, which in turn represents the motor steering ribosome ratcheting, ultimately placing fMet-tRNA in the productive P-site position and triggering the dissociation of IF2 [6,17,26]. An alternative view is that the ratcheting movement of the ribosome, which can occur also in the absence of factors [27,28,29], is thermally driven [30,31,32], and that the irreversible hydrolysis of GTP confers a directionality to the process, acting as a pawl in the Brownian ratcheting, and is absolutely essential only for the removal of the “toxic” γ-phosphate of GTP. In this article, we show that an IF2 mutant totally lacking GTPase activity and with a strongly reduced affinity for fMet-tRNA can suppress the dominant lethal phenotype associated with the GTPase inactivation and can promote the formation of a productive 70S EC and faithful mRNA translation. These findings are fully compatible with the second of the two models outlined above.

## 2. Results

### 2.1. Dominant Lethal Phenotype of IF2 H448E Mutant and Suppression of Lethality

**Figure 1:**
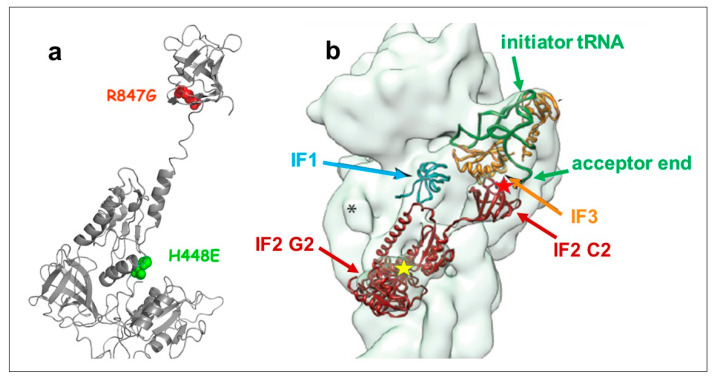
Location of the amino acid substitutions within the IF2 molecule. (**a**) Location of the His448 (green) mutation in the G2 domain and the Arg847 mutation (red) in the C2 domain of *E. coli* IF2. Because the C2 domain was not visible in the X-ray structure of bacterial IF2 [3,24], the positions of these amino acids are shown within the 3D structure of aIF5B, the archaeal IF2 homologue of IF2 [34]; (**b**) location of His 448 (yellow star) and Arg847 (red star) in the IF2 molecule (brown) within a 30S initiation complex with respect to the location of the acceptor end of initiator tRNA (green) and of initiation factors IF1 (cyan) and IF3 (orange). The positions of the 30S (gray) ligands are derived from cryo-EM reconstitutions [14].

Bacterial initiation factor IF2 contains a His residue at position 448 in *E. coli* and 301 in *Geobacillus stearothermophilus*. These residues, which correspond to H84 of EF-Tu, are located in the IF2 G2 domain [4] and occupy the last position in the conserved DXXGH sequence of the second of the three consensus elements which characterize GTP-binding proteins [33]. The position of the His448 residue within the structure of the factor is shown in Figure 1a,b and Figure S1 in Supplementary Materials. 

Histidine 84 of elongation factor EF-Tu was mutagenized in *E. coli* [35,36] and *Thermus thermophilus* [37] and, depending upon the nature of the substitution, were shown to have unaltered GTP binding, but a drastically reduced GTPase activity. Replacement with Glu, Gln, and Gly of the corresponding His448 in *E. coli* IF2 generated mutants also defective in GTP hydrolysis and displaying a dominant lethal phenotype [38,39]. Identical results were obtained upon substitution of His301 of *G. stearothermophilus* IF2 with Ser, Leu, and Tyr, whereas substitutions with and Gln and Arg did not result in a dominant lethal phenotype in spite of the loss of GTPase activity displayed upon His substitution with Arg [40,41].

**Figure 2:**
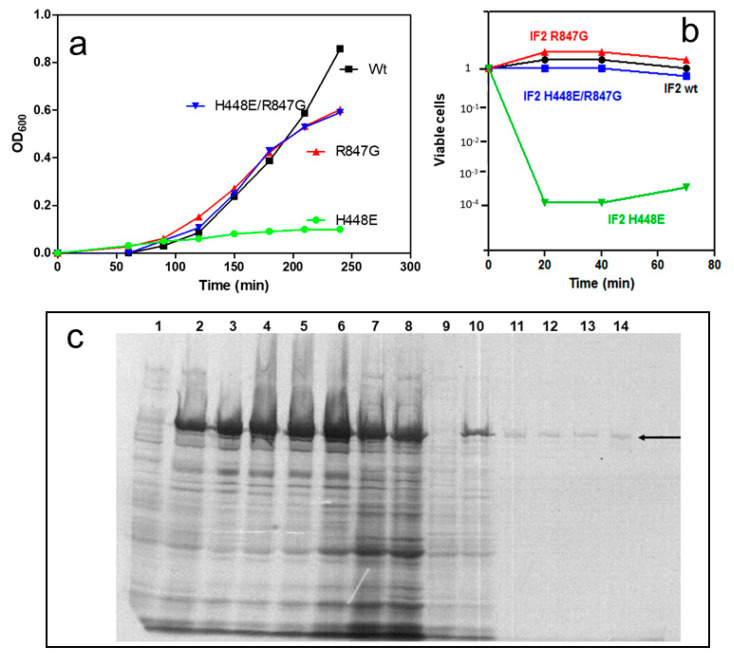
Dominant lethal phenotype of IF2 H448E and lethality suppression by R847G substitution. (**a**) *E. coli* cell cultures were grown to OD_600_ ~ 0.5 and induced (time 0) by addition of 1 mM IPTG to produce IF2 wt (black), IF2 R847G (red), IF2 H448E/R847G (blue) and IF2 H448E (green). After induction, the growth was followed by measuring the OD_600_ at the times indicated in the abscissa. The initial optical densities of the various cultures at the time of induction (approximately OD_600_ = 0.5) were normalized and indicated = 0; (**b**) at the indicated times after induction, samples were withdrawn for quantification of viable cells. The numbers of viable cells before induction (time = 0) were normalized and indicated = 1; (**c**) in vivo protein pulse–chase labeling with [^35^S]methionine. The cultures of *E. coli* UT5600 cells growing at 30 °C and carrying plasmid pXP101 (*infB* wt) (lanes 1–8) or pXP101*infB* H448 (lines 9–14) were given 3 min pulses of [^35^S]methionine followed by a chase with excess non-radioactive methionine before (lanes 1 and 9) and after increasing the temperature to 42 °C to induce IF2 expression (lanes 2, 3, 4, 5, 6, 10, 11, 12, 13, and 14). After 15 min at 42 °C, the temperature of the culture was lowered to 37 °C. The pulses were given at: −4′–0′ before induction (lanes 1, 9); 0′–4′ (lanes 2,10); 3′–6′ (lanes 3,11); 5′–8′ (lanes 4,12); 7′–10′ (lanes 5,13); 11′–14′ (lanes 6,14) after thermal induction; 5′–8′ (lane 7); 15′–18′ (lane 8) after lowering the temperature to 37 °C. The samples were processed and subjected to electrophoresis, as described in Materials and Methods. The arrow indicates the position where IF2 migrates, as determined by subjecting to electrophoresis a sample of purified non-radioactive factor loaded on a lane which was not autographed.

The dominant lethal phenotype occurring when His448 of *E. coli* IF2 was substituted with Glu in an extra chromosomal copy of *infB* can be observed in the experiments shown in Figure 2. After induction of expression of this mutated gene, despite the presence of a wild type copy of *infB* in the chromosome, the cells stopped growing (Figure 2a) and a rapid and drastic reduction of the number of viable cells ensued (Figure 2b).

Furthermore, electrophoretic analysis of extracts of cells [^35^S]-labeled at the time of induction revealed that a considerable amount of IF2 wt had been overexpressed by the control cells expressing exclusively *infB* wt, whereas de novo synthesized IF2 could not be detected in the cells expressing the mutated *infB*, aside from a tiny amount seen in the very initial times after the induction (Figure 2c). In addition, after the induction of the IF2 H448E expression, the amount of bulk cellular proteins synthesized appeared drastically reduced in comparison with the control, indicating that bulk translation is blocked by the presence of even minute amounts of the mutated IF2 (Figure 2c).

It had been shown that the dominant lethal phenotype caused by the His448 or His 301 substitutions can be suppressed by three secondary mutations in the G2 and C3 domains of IF2, which reduce the ribosomal affinity of the factor [40,41]. 

Upon introducing random mutations in C2, the fMet-tRNA binding domain of IF2 [42,43], we isolated a number of more or less efficient suppressors of the dominant–lethal phenotype caused by the H448E substitution. Among these, a particularly effective suppressor of lethality proved to be a mutant carrying an R847G substitution, in addition to the H448E substitution. 

The synthesis of this IF2 H448E/R847G doubly mutated IF2, like that of IF2 wt and IF2 R847G, did not inhibit cell growth, unlike the case of IF2 H448E (Figure 2a) and did not reduce the number of viable cells (Figure 2b). The localization of the R847 residue within the IF2 molecule is shown in Figure 1. Thus, the IF2 H448E/R847G double mutant as well as the IF2 R847G and the IF2 H448E single mutants were selected to pursue further characterization of the mechanisms responsible for the suppression. 

To bypasss the dominant lethal phenotype of IF2 H448E, we obtained large amounts of the IF2 H448E molecules upon expressing in yeast cells the corresponding *infB* gene. To ensure a homogeneous method of recombinant protein production and to avoid the contamination of the mutated IF2 molecules with IF2 wt, the same heterologous expression was also used to produce IF2 H448E/R847G and IF2 R847G.

### 2.2. Both H448E and H448E/R847G IF2 Mutants Are Inactive in GTP Hydrolysis

**Figure 3:**
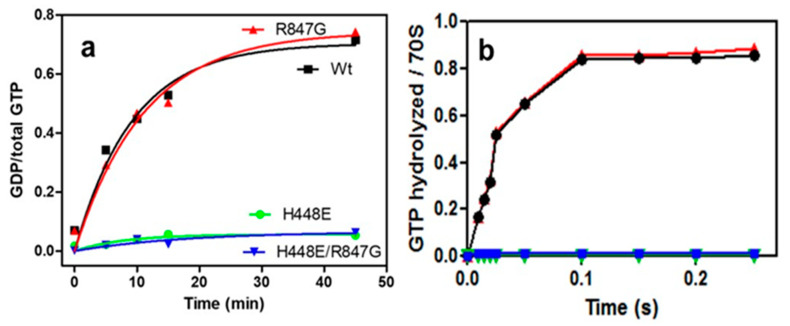
GTPase activity of IF2 wt and IF2 mutants. The kinetics of GTPase activities of IF2 wt (black), IF2 R847G (red), IF2 H448E/R847G (blue), and IF2 H448E (green) were analyzed (**a**) manually upon incubation at 37 °C for the indicated times in multiple turnover mode in the presence of ligand-free 70S ribosomes and (**b**) with a quench–flow apparatus in a single round hydrolysis mode upon formation of complete 70S initiation complexes. Experimental details can be found in Materials and Methods.

In agreement with published data [38,39], the IF2 H448E proved to be totally inactive in ribosome-dependent GTP hydrolysis; the doubly mutated IF2 H448E/R847G factor was also inactive in GTP hydrolysis, suggesting that the suppression of the dominant lethal phenotype is not due to the restoration of the GTPase activity of IF2. By contrast, IF2 R847G had wild type activity in GTP hydrolysis (Figure 3).

The failure of IF2 H448E and IF2 H448E/R847G to hydrolyze GTP does not stem from a defective binding of the guanosine nucleotide. In fact, binding experiments carried out with mant-GTP showed that upon incubation of this ligand with IF2 wt and with all IF2 mutants there is an increase of the amplitude of mant-GTP fluorescence. These experiments also suggested that the structures of the IF2 molecules bearing the His448E have a somewhat altered structure (see Figure S2a,b,c in Supplementary Materials). 

### 2.3. Formation of 30S and 70S Initiation Complexes in the Presence of IF2 Mutants

**Figure 4:**
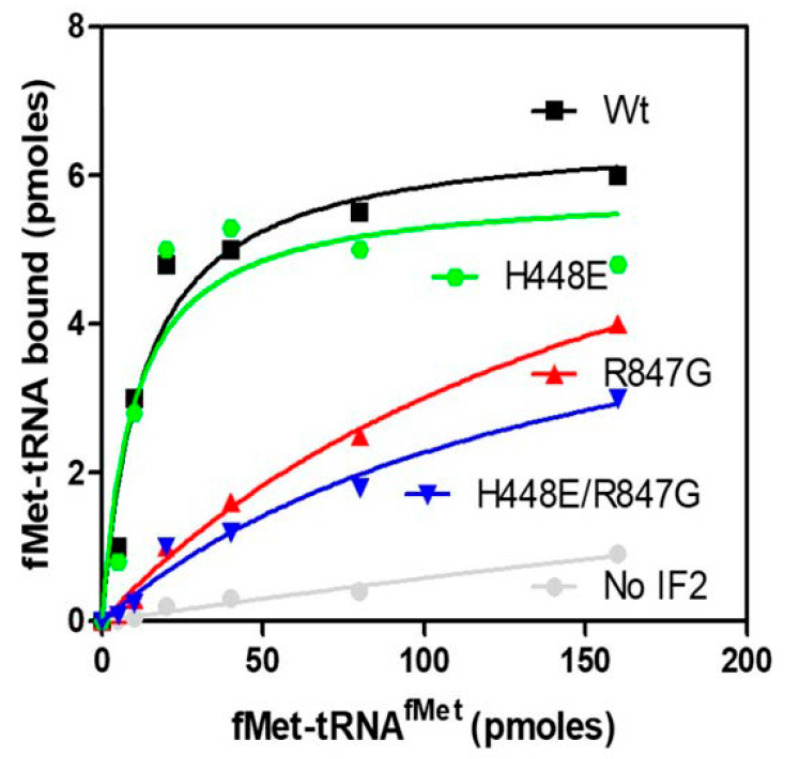
Formation of 30S IC in the presence of IF2 wt and IF2 mutants. The amounts of fMet-tRNA bound to 30S ribosomal subunits programmed with 022 mRNA in the presence of IF1, IF3 and of purified IF2 wt (black), IF2 R847G (red), IF2 H448E/R847G (blue), and IF2 H448E (green) were measured after 15 min incubation at 37 °C as a function of increasing amounts of initiator tRNA offered. The experimental conditions are the same as indicated in the legend of Figure 5.

**Figure 5:**
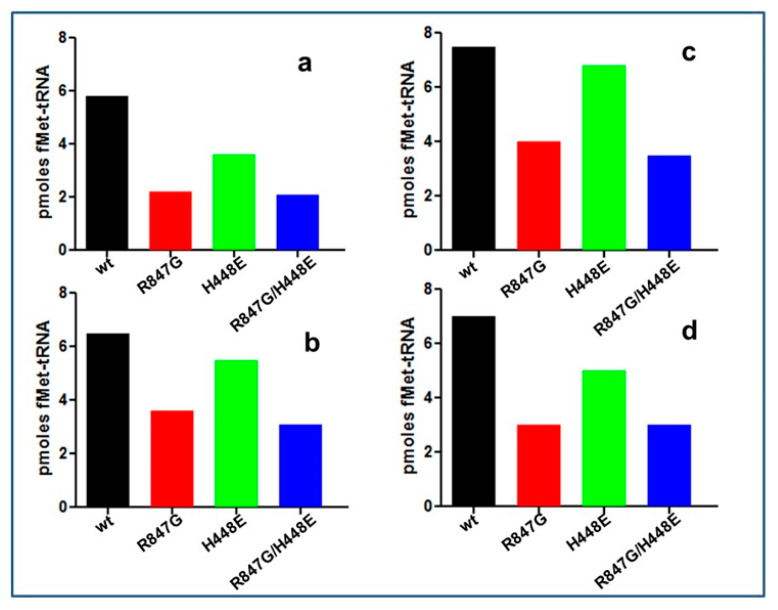
Levels of 30S IC (panels (**a**,**c**)) and 70S IC (panels (**b**,**d**)) formed in the presence of IF2 wt and IF2 mutants. The incubation mixtures contained, in 40 μL in Buffer D, 1mM GTP, 10 pmol each of 30S subunits or 70S monomers, initiation factors IF1 and IF3, f [^3^H]Met-tRNA, 022mRNA. In addition, the reaction mixtures contained 10 pmol (panels (**a**,**b**)) or 60 pmol (panels c and d) of purified IF2 wt (black), IF2 R847G (red), IF2 H448E/R847G (blue), and IF2 H448E (green). After 15 min incubation at 37 °C, the amounts of fMet-tRNA bound in the 30S (**a**,**c**) or 70S (**b**,**d**)) initiation complexes were determined by Millipore filtration. The levels of fMet-tRNA bound to ribosome in the absence of IF2 have been subtracted.

The capacity of IF2 mutants to promote the binding of fMet-tRNA to mRNA-programmed 30S ribosomal subunits in the presence of IF1 and IF3, thereby forming 30S ICs, was analyzed as a function of increasing amounts of initiator tRNA offered (Figure 4). As seen from the results shown in the figure, the two mutants in which the R847G mutation was present (i.e., IF2 R847G and H448E/R847G) displayed a reduced activity compared to IF2 wt. In light of the fact that IF2 H448E promotes the formation of the complex with essentially the same efficiency displayed by IF2 wt, it can be concluded that the R847G mutation is the cause of the reduced efficiency. Furthermore, since the reduced activity of the mutants is particularly marked at low fMet-tRNA input, it can be surmised that the reduced activity likely stems from a reduced affinity for the initiator tRNA. In another experiment, the efficiencies of the IF2 mutants to promote 30S IC and 70S IC formation were compared. The results confirmed that, compared to IF2 wt, both IF2 R847G and H448E/R847G have a markedly reduced activity in supporting fMet-tRNA binding to 30S subunits, whereas IF2 H448E had almost the same activity as IF2 wt (Figure 5a). Remarkably, the ability to promote fMet-tRNA binding to 70S ribosomes was reduced by approximately 50% compared to 30S binding with all IF2 mutants (Figure 5b). This finding indicates that the 30S IC formed in the presence of the IF2 mutants have a somewhat defective, non-canonical orientations/structures which may impair efficient joining of the 50S to form the 70S IC.

### 2.4. The IF2 Mutants Bearing the R847G Substitution Have a Reduced Affinity for fMet-tRNA 

**Figure 6:**
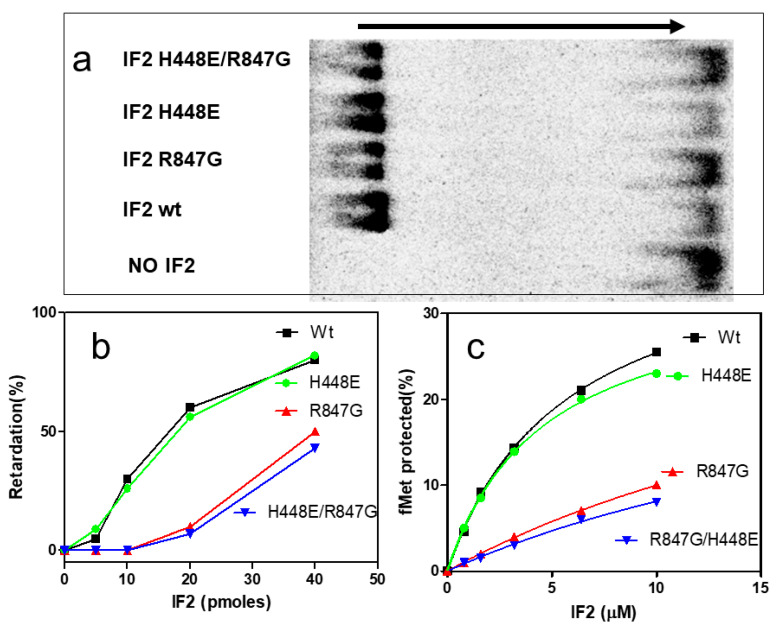
Affinity of IF2 wt and IF2 mutants for fMet-tRNA. The interaction between fMet-tRNA and IF2 wt or IF2 mutants and was analyzed by gel shift electrophoresis and by fMet-tRNA protection from hydrolysis by RNase A. (**a**) autoradiography of the electrophoretic migration of f[^35^S]Met-tRNA preincubated with or without IF2 wt or IF2 mutants. The direction of migration is from left to right, as indicated by the arrow. In the absence of IF2, free f[^35^S]Met-tRNA migrates as a single band to the right, whereas in the presence of IF2 wt and of IF2 H448E, the amount of free f[^35^S]Met-tRNA is strongly reduced while the bulk of the initiator tRNA is retarded and found in the band at the extreme left. In the presence of IF2 R847G and H448E/R847G, the electrophoretic retardation of fMet-RNA is strongly reduced; (**b**) quantification of the extent of electrophoretic retardation of f[^35^S]Met-tRNA upon incubation with the increasing amounts of purified IF2 wt (black), IF2 R847G (red), IF2 H448E/R847G (blue), and IF2 H448E (green) indicated in the abscissa; (**c**) extent of fMet-RNA protected from RNase hydrolysis upon incubation with the indicated amounts of IF2 wt (black), IF2 R847G (red), IF2 H448E/R847G (blue), and IF2 H448E. Further details can be found in Section 4.

The premise that the two IF2 mutants bearing the R847G substitution have a reduced affinity for fMet-tRNA, as suggested by the results of the IF2-promoted 30S IC formation, was tested by gel shift and by fMet-tRNA protection experiments. When free fMet-tRNA is subjected to agarose gel electrophoresis, the molecule migrates as a single, fast moving band. However, upon incubation with IF2 wt, a large proportion of the initiator tRNA migrates more slowly, being present in a band found in a position closer to the origin; a similar behavior is observed also when fMet-tRNA is preincubated with IF2 H448E (Figure 6a). 

However, when the initiator tRNA was pre-incubated with the IF2 mutants bearing the R847G substitution, the amount of electrophoretically retarded fMet-tRNA was strongly reduced compared to IF2 wt. The extent of fMet-tRNA retardation, quantified as a function of the amount of IF2 molecules present in the pre-incubation mixture, is presented in Figure 6b. As seen from the figure, fMet-tRNA is efficiently retarded by IF2 wt and IF2 H448E already in the presence of low amounts of factor, whereas under the same conditions, there was hardly any retardation upon incubation with the two mutants bearing the R847 substitution. Only about 10% of the initiator tRNA was electrophoretically retarded in the presence of 20 pmol of these IF2 mutants, whereas the same amounts of IF2 wt and IF2 H448E caused the retardation of about 70% of the tRNA.

The interaction between IF2 and fMet-tRNA was also investigated by analyzing the capacity of the different types of IF2 molecules to protect initiator tRNA from the hydrolytic activity of RNase. In full agreement with the results of the gel shift experiments, this approach also demonstrated that IF2 wt and IF2 H448E bind fMet-tRNA better than IF2 R847G and IF2 H448E/R847G as they can protect much more efficiently the 3′ end of initiator tRNA from enzymatic hydrolysis (Figure 6c). Taken together, these results provide an explanation for the reduced capacity of IF2 R847G and IF2 H448E/R847G to promote 30S IC and 70S IC formation (Figure 4 and Figure 5) and indicate that the suppression by IF2 H448E/R847G of the dominant lethal phenotype caused by the H448E substitution can likely be attributed to the reduced affinity for fMet-tRNA of the double mutant.

### 2.5. “Initiation Dipeptide” Formation and mRNA Translation with IF2 Mutants

The dominant lethal phenotype of the H448E mutation is caused by a general blockage of cellular protein synthesis even in the presence of IF2 wt in the cells (Figure 2c). It is possible that the mutated IF2 molecules enter the translational initiation cycle, but, being unable to hydrolyze GTP and thereby to dissociate from the ribosomes, they remain stuck and block the progress of all ribosomes into the elongation phase of translation. Therefore, a release of this block by weakening the interaction of the factor with the ribosomes must be the mechanism by which the secondary mutation of R847 causes the suppression of lethality.

**Figure 7:**
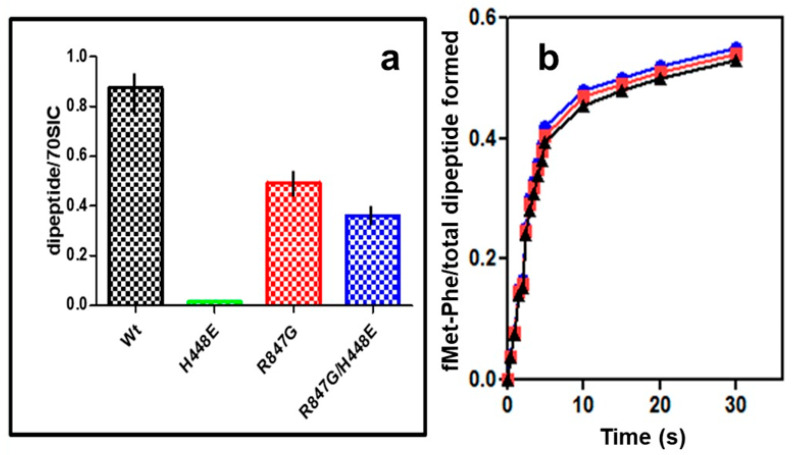
Initiation dipeptide formation in the presence of IF2 wt and IF2 mutants. The f[^3^H]Met-[^14^C]Phe initiation dipeptide was formed by mixing and incubating equal volumes of EF-Tu •GTP• [^14^C]Phe-tRNA ternary complex and 70S IC formed in the presence of IF2 wt (black), IF2 R847G (red), IF2 H448E/R847G (blue), and IF2 H448E (green) and f[^3^H]Met-tRNA. The experimental details are given in Materials and methods. (**a**) The level of dipeptide formed in the presence of IF2 wt or IF2 mutants after 20 min incubation at 37 °C is expressed as the ratio of dipeptide formed vs. amount of initiator fMet-tRNA present in the corresponding 70S IC; (**b**) kinetics of initiation dipeptides formation by 70S IC complexes formed in the presence of IF2 wt or IF2 mutants. No dipeptide was formed in the absence of IF2.

The successful suppression of lethality does not tell us anything about the activity of the doubly mutated IF2 which lacks GTPase activity. Therefore, the capacity of the IF2 mutants to support maturation of the 70S IC into a productive 70S EC was assessed by measuring their capacity to promote formation of the first peptide bond between P-site bound fMet-tRNA and the first incoming aminoacyl-tRNA bound to the ribosomal A site in response to the second mRNA codon. In Figure 7a, the level of the fMet-Phe “initiation dipeptide” formed in the presence of the mutant IF2 molecules is expressed as percent of the fMet-tRNA molecules present in the 70S IC, which is about 40% the level obtained with IF2 wt, as seen in Figure 5b. In addition, in the cases of 70S ICs formed in the presence of IF2 R847G and IF2 H448E/R847G, the efficiency by which the 70S-bound fMet-tRNA participates in “initiation dipeptide” formation is only 40–50% compared to IF2 wt (Figure 7a). Thus, the overall level of “initiation dipeptide” formed in the presence of the IF2 mutants bearing the R847G substitution is much lower compared to the level obtained with IF2 wt. Nevertheless, a substantial amount of the fMet-Phe “initiation dipeptide” is formed also in the presence of IF2 R847G and IF2 H448E/R847G, whereas IF2 H448E was completely inactive (Figure 7a). Furthermore, the kinetics at which the “initiation dipeptide” formation approaches completion (i.e., the level attained after 15 min incubation) are indistinguishable for 70S IC containing IF2 wt or IF2 mutants bearing the R847G substitution with a k_app_ = 0.45 ± 0.05 (Figure 7b) for everyone of them.

**Figure 8:**
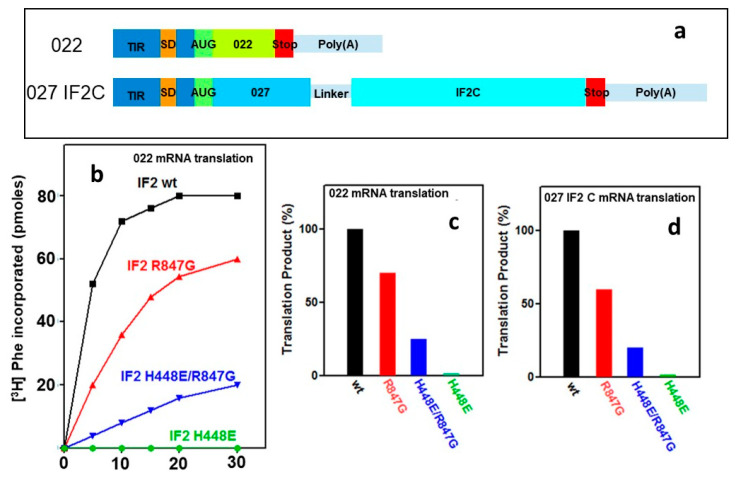
mRNA translation in the presence of IF2 wt and IF2 mutants. (**a**) Schematic representation of the mRNAs used in the translation tests. The corresponding sequences are reported in Supplementary Materials (Figure S3a,b). Translation of mRNA in cell free systems containing IF2 wt (black), IF2 R847G (red), IF2 H448E/R847G (blue), and IF2 H448E (green); (**b**) Time course of [^3^H] phenylalanine incorporation into acid-insoluble product under the direction of model 022mRNA [45]. The incubation was carried out at 37 °C for the indicated times or for 45 min (panel (**c**)); (**d**) the level of 027IF2C mRNA translational was quantified by ELISA using monoclonal antibodies directed against IF2C, as described [45]. In panels (**c**,**d**), the translational level obtained in the presence of the IF2 mutants is expressed as % of the level obtained with IF2 wt. No detectable translation was observed in the absence of IF2.

The capacity of the IF2 H448E/R847G double mutant to promote the assembly of a 70S IC active in initiation dipeptide formation suggests that this mutant, in spite of lacking GTPase activity, might also be capable of promoting mRNA translation. This premise was tested carrying out in vitro translation experiments using cell free systems programmed with the model 022 mRNA and containing IF2 wt or IF2 mutants. The time course of translation showed that, as expected, the system was fully active when IF2 wt was present and totally inactive in the presence of IF2 H448E. On the other hand, a substantial level of translation was obtained in the presence of IF2 R847G and in the presence of the IF2 H448E/R847G double mutant, albeit to a lower extent compared to wt (Figure 8a). Compared to the translation level attained by the system containing IF2 wt, the levels obtained in the presence of IF2 R847G and IF2 H448E/R847G were 70% and 25%, respectively (Figure 8b). This reduced activity is likely due to the reduced affinity for the initiator tRNA of the IF2 molecules bearing the R847G mutation and by an unfavourable modification of the IF2 structure caused by the H448 substitution, as suggested by the binding experiments with mant-GTP (Figure S1) and by the disparate phenotypes resulting from different mutations of this Histidine [44].

An additional test was carried out to ascertain that the product synthesized in the presence of the IF2 mutants does not consist of random amino acids incorporated into an acid insoluble product but represents instead a faithful translation of the mRNA-encoded protein. For this purpose, protein synthesis tests were repeated using cell free systems programmed with an mRNA encoding a natural protein, namely the C domain of IF2, whose in vitro product can be detected and quantified immunologically [45]. The results of these experiments confirmed that mRNA translation can occur in the presence of the IF2 factors carrying the R847G mutation and that translation faithfully yields a product recognized by monoclonal antibodies directed against the protein encoded by the mRNA used to program the synthesis (Figure 8c). As in the previous case (Figure 8a,b), no synthesis occurred in the presence of the IF2 molecule carrying a single mutation of the His448 residue, but a sizable level of translation occurred when also a second mutation of R847 was present.

## 3. Discussion

The substitution of the conserved Histidine located at the end of the second (DXXGH) of the three consensus elements that characterize G2, the guanosine nucleotide binding domain of IF2, causes the complete inactivation of the GTPase activity of the factor. In turn, the loss of this hydrolytic activity results in IF2 molecules which are unable to dissociate from the ribosome, block protein synthesis in the cell, even in the presence of IF2 wt, thus determining a dominant lethal phenotype [38,39,40,41]. It has been shown that the toxic effect of the GTPase-defective IF2 mutants can be overcome by secondary mutations (i.e., E571K in *E. coli* and E424K in *G. stearothermophilus*) which reduce the affinity of the factor for the ribosome and act as suppressors of lethality [40,41].

The data presented here demonstrate that not only a weakening of the ribosomal affinity of IF2, but also weakening the interaction between IF2 and fMet-tRNA is able to suppress the dominant lethal phenotype caused by GTPase inactivation.

Taken together, these findings confirm the premise that the toxic effect of the GTPase defective IF2 is due to the failure of the factor to dissociate from the ribosomes causing a generalized block of protein synthesis. Accordingly, any IF2 modification which loosens the ties which keep the factor bound to ribosomes can suppress the toxic effect and, consequently, also the dominant lethal phenotype caused by the His mutation.

**Figure 9:**
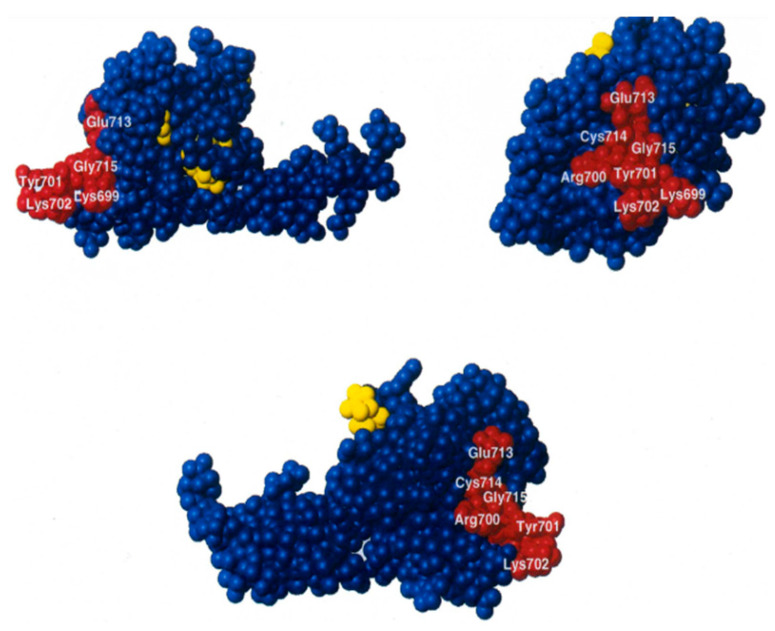
Location of the amino acid residues essential (red) and non-essential (yellow) for the IF2-fMet-tRNA interaction identified by mutagenesis shown within different views the 3D structure of *G. stearothermophilus* IF2 C2 (blue) determined by NMR spectroscopy [46]. Arg700 corresponds to *E. coli* Arg847. Figure taken from [43].

The R847G mutation which suppresses lethality is located in the fMet-tRNA binding region of the C2 domain of the factor [42,43,46,47] (Figure 1 and Figure 9) and strongly reduces the affinity of IF2 for the initiator tRNA, as shown by the electrophoretic gel shift experiment and by the reduced protection of fMet-tRNA from RNase hydrolysis (Figure 6a–c).

In this connection, it should be recalled that the interaction between IF2 C2 and the 3′ end of initiator tRNA represents the main and last anchor which keeps the factor ribosome-bound and sequesters the acceptor end of fMet-tRNA, thereby preventing initiation dipeptide formation. The reduced affinity for fMet-tRNA of IF2 molecules bearing the R847G mutation results in a reduced activity in promoting fMet-tRNA binding to both 30S subunits and 70S monomers to form 30S IC and 70S IC, respectively (Figure 4 and Figure 5a,b) and, consequently, in reduced levels of initiation dipeptide formed (Figure 7) and mRNA translation (Figure 8). However, the relevant finding of the present study is that the lethality suppressor mutant IF2 H448E/R847G, which is totally inactive in GTP hydrolysis like the dominant lethal mutant IF2 H448E, is capable of promoting initiation dipeptide formation and faithful mRNA translation. From this finding, it can be concluded that GTP hydrolysis is not a prerequisite for these activities, in full agreement with the report showing that translation initiation, protein synthesis, and cell growth can occur at near-normal wild type rates in the absence of IF2-dependent GTP hydrolysis [41].

In light of this, it is necessary to question the function of IF2-dependent GTP hydrolysis during translation initiation. Upon 30S IC formation, the presence of IF2 GTP accelerates the docking of the 50S subunit, prompting the subunits to assume a semi-rotated orientation which facilitates formation and stabilization of inter-subunit bridges. Subunit association activates the immediate IF2-dependent hydrolysis of GTP, which occurs at a rate of ∼30 s^−1^. After the hydrolysis, IF2 GDP Pi remains ribosome-bound and release of Pi is delayed, being nearly one order of magnitude slower than the hydrolysis, and occurs at a later stage, together with the dissociation of fMet-tRNA from IF2 [6,16,20]. In the presence of IF2 GTP, IF2 GDPCP, or IF2 GDP Pi, the γ-phosphate interacts with switch II of IF2, which adopts an ordered and helical conformation, whereas it becomes disordered when the γ-phosphate is lost, as in IF2 GDP. The structural change occurring in switch II causes IF2 to assume a different conformation with consequent disruption of the IF2-ribosome interactions which stabilize the semi-rotated inter-subunit orientation of the 70S. As a result, the 30S subunit undergoes a ~3° reverse rotation which allows the 70S to assume a non-rotated inter-subunit orientation. [9,14]. Meanwhile, contact between IF2 C2 and fMet-tRNA_fMet_ is disrupted, allowing the central domain and 3′ end of the initiator tRNA to move ~28 Å and ~22 Å, respectively, from the 70S P/I position observed in the 70S IC to the P/P configuration observed in the 70S EC [8,9,10,14,15]. Loosening of the IF2 C2-fMet-tRNA interaction frees both IF2, which can dissociate from the ribosome, and the initiator tRNA, which can participate in initiation dipeptide formation [16,20].

Thus, a key function of GTP hydrolysis is to predispose the elimination of the γ-phosphate, whose presence blocks the conformational change of IF2 which allows the dissociation of the factor from the ribosome [6,7,14,15,39,41,43,47]. The failure of the IF2 mutants lacking GTPase activity (i.e., IF2 H448E) to dissociate from the ribosome causes a generalized block of translation and a dominant lethal phenotype. However, when the mutation which inactivates the GTPase is present together with a secondary mutation which weakens the IF2 affinity for the ribosome, either directly [40,41] or indirectly, as in the present case of IF2 H448E/R847G, the lethal phenotype is suppressed.

GTPase-defective IF2 H448E/R847G supports a detectable level of initiation dipeptide formation (Figure 7) and faithful mRNA translation (Figure 8), and cells exclusively expressing an IF2 molecule completely inactive in GTPase activity can grow and divide at a near normal rate [41].

These findings indicate that the energy liberated by IF2-dependent GTP hydrolysis is not required for translation and for cell survival. This conclusion is fully compatible with the premise that the conformational changes of ribosomes and IF2, occurring during the maturation of the 70S IC into 70S EC, like ribosome ratcheting during translocation, are driven by Brownian thermal fluctuations and the GTP hydrolysis represents an irreversible chemical step which confers directedness to the process [27,30,31,32].

## 4. Materials and Methods

### 4.1. Buffers

- Buffer A: 10 mM Tris-HCl, pH 7.7, 60 mM NH_4_Cl, 5 mM Mg Acetate_2_, 5 mM β-mercaptoethanol, 0.2 mM PMSF.
- Buffer B: 50 mM Tris-HCl, (pH 7.5), 80 mM NH_4_Cl, 30 mM KCl, 7 mM MgCl_2_,1 mM DTT.
- Buffer C: 50 mM HEPES/KOH (pH 7.5), 10 mM MgCl_2_, 50 mM NH_4_Cl.
- Buffer D: 20 mM Tris-HCl (pH 7.7), 7 mM Mg Acetate_2_, 60 mM NH_4_Cl, 1 mM DTT.
- Buffer E: 150 mM Tris-HCl (pH 7.5), 75 mM NH_4_Cl, 10 mM MgCl_2_, 5 mM β-mercaptoethanol

### 4.2. General Preparations

*E. coli* 70S, 30S, and 50S ribosomal subunits, S100 post-ribosomal supernatant, f[^35^S]Met-tRNA_fMet_, 022, and 027IF2Cp(A) mRNAs were prepared as described [45,48]. Initiation factors IF1, IF3, and IF2 wt and mutants were purified as described [49]. The S100 post-ribosomal supernatant was incubated with 3 µM 30S subunits and centrifuged 1 h at 100K × rpm (Sorvall RC M120 GX ultracentrifuge, S100AT3-205 rotor) to remove possible trace amounts of IF2 wt whose complete absence was ascertained by Western blotting using monoclonal antibodies directed against IF2 C2.

### 4.3. Strains, Growth Media, and Plasmid Constructs

Mutant alleles of *infB* were constructed by subjecting wild type *infB*, carried by plasmid pXP101 [50], to site directed mutagenesis [51] to obtain pXP101*infB H448E*, pXP101*infB H448E*/*R847G*, and pXP101*infB R847G* encoding IF2 H448E, H448E/R847G, and IF2 R847G, respectively. These plasmids were transformed into *E. coli* UT5600 [52] carrying λcI^ts^ repressor (expressed by plasmid pcI*857*) so as to bring the expression of wild type and mutant *infB* genes under control of the thermo-inducible pL and pR promoters.

### 4.4. Heterologous Expression of IF2 wt and Mutants in Yeast

The expression system of *Saccharomyces cerevisiae* carrying pEMBLyex2 + *E coli infB* (wild-type and mutants) has been described [53]. Wild type and mutant *infB* genes were placed under the control of a galactose inducible promoter and cloned in the pEMBLyex2 shuttle vector [53] which was transferred to the *S. cerevisiae* strain S150-PA1 (MAT*a leu2,3-112 trp1-289 his3-Δ1 ura3-52 lys2::TRP1::PDI*) which was grown either in YP rich medium (1% yeast extract, 2% peptone, 0.3% KH_2_PO_4_) supplemented with 2% glucose (named YPD) or in minimal medium (0.67% yeast nitrogen base w/o amino acids (Difco), 2% glucose, supplemented with 0.005% of lysine, histidine, and leucine).

### 4.5. Transformation of S. cerevisiae

*S. cerevisiae* S150-PA1 was transformed using the LiCl method [54]. The cells were grown in YPD medium at 30 °C to A_640_ ≅ 0.5, harvested by centrifugation for 7 min at 1.1K × rpm, resuspended in 1/5 volume of TE (10 mM Tris-HCl, pH 8.0 and 1 mM EDTA) and centrifuged again. The pellets were resuspended in TE containing 0.1 mM LiCl (1/30 of starting volume) and left on ice for 3–8 h. Approximately 20 μL of DNA solution (≥5 μg of plasmid DNA) were added to 200 μL competent cells which were incubated for 30 min at room temperature. After addition of 1.5 mL of 40% PEG 4000 in 0.1 M LiCl in TE, the cells were gently mixed and kept 1 h at room temperature then heat shocked at 42 °C for 15 min and centrifuged at 0.5K × rpm in SA600 rotor (Sorvall). The cell pellets were washed with 2 mL of sterile H_2_O (without resuspending the cells) to remove PEG and the cells were resuspended in 200 μL of H_2_O and the transformants selected on minimal medium plates containing histidine, leucine, and lysine.

### 4.6. Heterologous Expression of IF2 wt and Mutants

The yeast cells were grown at 30 °C in minimal medium to an A_640_ = 4–5, and overexpression was induced by addition of 3 volumes of GALYP medium (YP medium containing 4% galactose) followed by incubation for 5 days in a shaking water bath at 30 °C. For the IF2 purification, approximately 30 g of *S. cerevisiae* cells were ground in a mortar with Glass beads (Sigma 425–600 microns) 1 g/g of cells and the resulting cell paste resuspended with 30 mL of ice-cold Buffer A and centrifuged 1 h at 30K × rpm in a SA-600 (Sorvall) rotor. The supernatant, to which NH_4_Cl was added up to 1 M concentration, was centrifuged at 100K × rpm in a 50Ti Beckman rotor, and the resulting supernatant further processed for IF2 purification, as previously described [49].

### 4.7. Pulse–Chase Experiment

*E. coli* UT5600 cells carrying pXP101 (*infB* wt) or pXP101*infB H448E*, pXP101*infB H448E*/*R847G* and pXP101*infB R847G* were grown at 30 °C in M9 minimal medium. When the culture reached an A_600_ ~ 0.5, the temperature was increased to 42 °C for 15 min and 750 μL aliquots were distributed into tubes; 2.25 μCi of [^35^S]methionine were added before the induction (−4 to 0 min) and at different times after the induction.

Each pulse lasted 3 min and was followed by a chase with 90 μL of 250 mM non-radioactive methionine and 30 s incubation before the samples were transferred to an ice bath. After 5 min centrifugation at 13K × rpm, the cells were resuspended in 0.9% NaCl (*w*/*v*), centrifuged again and dissolved in SDS loading buffer containing 1% β-mercaptoethanol, and subjected to electrophoresis on SDS-PAGE (7.5% acrylamide). At the end of the electrophoreses, the gels were dried and subjected to autoradiography.

### 4.8. GTPase Activity of IF2wt and Mutants

Multiple-turnover GTP hydrolysis: The incubation mixtures (250 μL of Buffer B) contained 50 pmol of IF2 wt or IF2 mutants and [α-^32^P] GTP (specific activity ~ 1000 cpm/pmol) and GTP hydrolysis was started by addition of 75 pmol of 30S and 50S *E. coli* ribosomal subunits. After Incubation at 37 °C for the indicated times, 50 μL samples were withdrawn from each mixture and the reactions quenched by addition of 50 μL of 1 M HClO_4_ and 3 mM KH_2_PO_4_. The amount of hydrolyzed GTP was determined essentially as described [55] upon separation of [α^32^P]GTP and [α^32^P]GDP by TLC on plastic-backed polyethyleneimine plates which were developed for 4 h in 1.2 M ammonium formate/0.8 M HCl. The areas corresponding to GDP and GTP were cut out, and the radioactivity therein quantitated by liquid scintillation counting.

Kinetics of single round GTP hydrolysis 30S initiation complexes were prepared in buffer B using 30S subunits (0.6 mM), 022 mRNA (1.8 mM), f [^3^H] Met-tRNA_fMet_ (0.9 mM), and [γ-^32^P]GTP (72 mM, 1000 dpm/pmol), 0.9-mM each of IF1, IF3, and IF2 wt or IF2 mutants. After incubation at 37 °C for 15 min, the complexes were stored on ice. One sample syringe of a Biologique SFM-400 quench flow apparatus (Figure S4 in Supplementary Materials) was filled with the solution containing 30S initiation complexes (final concentration 0.3 mM) and the other with a solution containing 0.3 mM (final concentration) of 50S subunits in the same buffer. After rapid mixing, reactions were quenched by 1-M HClO_4_ with 3-mM KH_2_PO_4_, and [^32^P] phosphate was determined by molybdate extraction into ethyl acetate, as described [56].

### 4.9. fMet-tRNA Protection from Ribonuclease Hydrolysis by IF2

The protection was assayed essentially as described [57]. Increasing amounts (as indicated in the figure) of IF2 wt or IF2 mutants were incubated 10 min on ice in 100 μL of Buffer E with 2.5 μM f[^14^C] Met-tRNA_fMet_; ribonuclease A (10 μg) was added at t = 0 and the incubation continued for 30 min when the reaction was stopped by addition of an excess of bulk tRNA and 10% (final concentration) of trichloroacetic acid (TCA). The percent protection of f[^14^C] Met-tRNA_fMet_ was quantified by measuring the level of the TCA-insoluble radioactivity at time 0 and at the end of the incubation.

### 4.10. Gel-Retardation Experiments

The experiments were carried out essentially as described [57]. f[^35^S]Met-tRNA_fMet_ (1 μM final concentration) was incubated in 20 μL of Buffer C alone or with a 10-fold excess IF2 wt or IF2 mutants. After 10 min at 37 °C, the samples were loaded on gels consisting of 10% (*w*/*w*) acrylamide, 0.275% (*w*/*w*) bisacrylamide in 20 mM MOPS/NaOH pH 7.5. The electrophoreses were run for 3 h at V100 at 20 °C in the same buffer. The gels were dried and subjected to autoradiography and the amount of material present in the band corresponding to free fMet-tRNA and to the IF2-retarded band was determined by densitometry.

### 4.11. IF2-Dependent Formation of 30S and 70S Initiation Complexes

The reaction mixtures (40 µL of Buffer D) contained 30 pmol each of *E. coli* 30S ribosomal subunits or 70S monomers, IF1, IF3, IF2 wt or IF2 mutants, 45 pmol of 022 mRNA, 0.5 mM GTP, and the indicated amounts of f[^3^H]Met-tRNA. After 20 min incubation at 37 °C, 30 µL were removed from each reaction and the amount of ribosome-bound fMet-tRNA was determined by filtration through nitrocellulose discs [45,48].

### 4.12. Preparation of EF-Tu-GTP-Phe-tRNA Ternary Complex

The ternary complex was formed in Buffer D incubating for 10 min at 37 °C 1 mM GTP, 3 mM PEP, 0.25 µg/mL PK, and 1 µM each of EF-Tu and [^14^C]Phe-tRNA.

### 4.13. Initiation Dipeptide Formation

Formation of f[^35^S]Met-[^14^C]Phe dipeptide was quantified both manually [45] and by quenched flow analysis [48]. 30S ICs were prepared in 40 µL Buffer D in the presence of IF2 wt or IF2 mutants, as described above. After 15 min incubation at 37 °C, 5 µL aliquots were withdrawn from each reaction mixture to measure by nitrocellulose filtration the amount of f[^3^H]Met-tRNA bound in each 30S IC. Aliquots of 30 µL each were mixed with an equal volume of Buffer D containing the EF-Tu•GTP•[^14^C]Phe-tRNA ternary complex and 50S subunits (0.3 μM final concentration). After 5 min incubation at 37 °C, the reaction was stopped by addition of 60 µL of 0.5 M KOH. After 15 min at 37 °C, the mixture was neutralized with acetic acid and centrifuged at 12K × rpm for 5 min. The dipeptide formed was analyzed by HPLC on a reverse phase (LiChrosorb RP-8, 5 mM-Merck) column with a linear (0–65%) acetonitrile gradient in 0.1% TFA and the radioactivity present in the individual chromatographic fractions determined by liquid scintillation counting.

The kinetics of initiation dipeptide formation was analyzed at 20 °C with a Biologique SFM-400 quench flow apparatus (Figure S4). One syringe was loaded with Buffer D containing 2 mM GTP, 0.3 μM 30S IC assembled with IF2 wt or IF2 mutants and 0.6 μM of EF-Tu•GTP•[^14^C]Phe-tRNA ternary complex. The other syringe was loaded with Buffer D containing 0.3 μM 50S subunit. At the times indicated in the figure the reaction was quenched with 0.2 M KOH. After 15 min at 37 °C, the quenched mixture was neutralized with acetic acid, centrifuged for 5 min at 12K × rpm, and the peptides analyzed by HPLC.

### 4.14. HPLC Analysis of fMet-Phe Dipeptide

The analysis was carried out by HPLC, using a LiChrosorb RP-8,HPLC column 5 μm particle size (Merck) in a Kontron apparatus. The HPLC system was run at 1 mL/min using an adapted convex gradient consisting of solvent A = 0.1% (*w*/*v*) trifluoroacetic acid (TFA)) and B = 65% (*v*/*v*) acetonitrile and 0.1% TFA. Fractions from 6 to 16 min were collected and the radioactivity therein measured in a liquid scintillation counter. The first peak was [^14^C]Phe and the second was f[^35^S]Met-[^14^C]Phe. After having identified by the simultaneous presence of both f[^35^S]Met and [^14^C]Phe the position at which the initiation dipeptide is eluted, for economical reasons only, f[^35^S]Met-tRNA and non radioactive Phe-tRNA were used in the following experiments.

### 4.15. mRNA Translation In Vitro

The reaction mixtures consisted of 30 µL Buffer D containing 2 mM ATP, 0.4 mM GTP, 10 mM PEP, 0.025 mg/mL PK, 0.12 mM 5 10-formyl-tetrahydrofolate, 4 µL of IF2-free S100 post-ribosomal supernatant (~80 µg of total protein), 1 µM each of 70S ribosome, IF1, IF3 and either IF2 wt or IF2 mutants, 3 µM of either 022 mRNA or 027IF2C mRNA [43], as indicated. The reaction mixtures also contained 0.2 mM of all amino acids but for the radioactive precursors (9 µM non-radioactive and 1 µM [^3^H] phenylalanine for 022 mRNA translation and 49 µM nonradioactive and 1 µM [^3^H] glycine for 027 IF2C mRNA translation). After 30 min at 37 °C, the level of translation was quantified from the amount of acid-insoluble radioactive precursor incorporated (for 022 mRNA) and from the immunological quantification of the product (for 027IF2C mRNA translation) using monoclonal antibodies directed against IF2C, as previously described [45].

## 5. Bullets

The interaction between the C2 domain of IF2 and the acceptor end of initiator tRNA anchors the factor to the ribosome.

A mutation which weakens the interaction between IF2 C2 and initiator tRNA suppresses the dominant lethal phenotype caused by the mutational inactivation of the GTPase activity of IF2.

The γ-phosphate of GTP is toxic insofar as it prevents IF2 from assuming the conformation which allows the dissociation of IF2 from the fMet-tRNA acceptor and the adjustment of the initiator tRNA in the productive P/P site of the ribosome.

GTP hydrolysis by IF2 is a prerequisite for the release of the toxic γ-phosphate of GTP, but the energy liberated by this reaction is not required for translation.

## Data Availability

Not applicable.

## Supplementary Materials

The following are available online at https://www.mdpi.com/article/10.3390/ijms222413238/s1.
