# Supplementary material for: Weakening the IF2-fMet-tRNA Interaction Suppresses the Lethal Phenotype Caused by GTPase Inactivation
Source: Int J Mol Sci. 2021 Dec 8;22(24):13238. doi: 10.3390/ijms222413238 (PMC8709274; doi:10.3390/ijms222413238)
Supplement: Supplementary file 1 [file ijms-22-13238-s001.zip › ijms-1465756-supplementary.pdf]

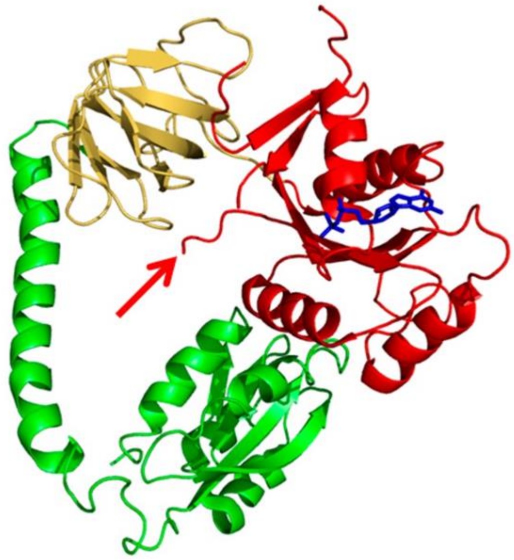

Figure S1 3D structure of *Thermus thermophilus* IF2 bound to GDP (blue). The IF2 domains are color-coded: G2 (red), G3 (yellow) and C1 (green). The position, of the His residue corresponding to *E. coli* His448, located at the tip of switch II, is indicated by the red arrow. The figure is from pdb file 4KJZ.

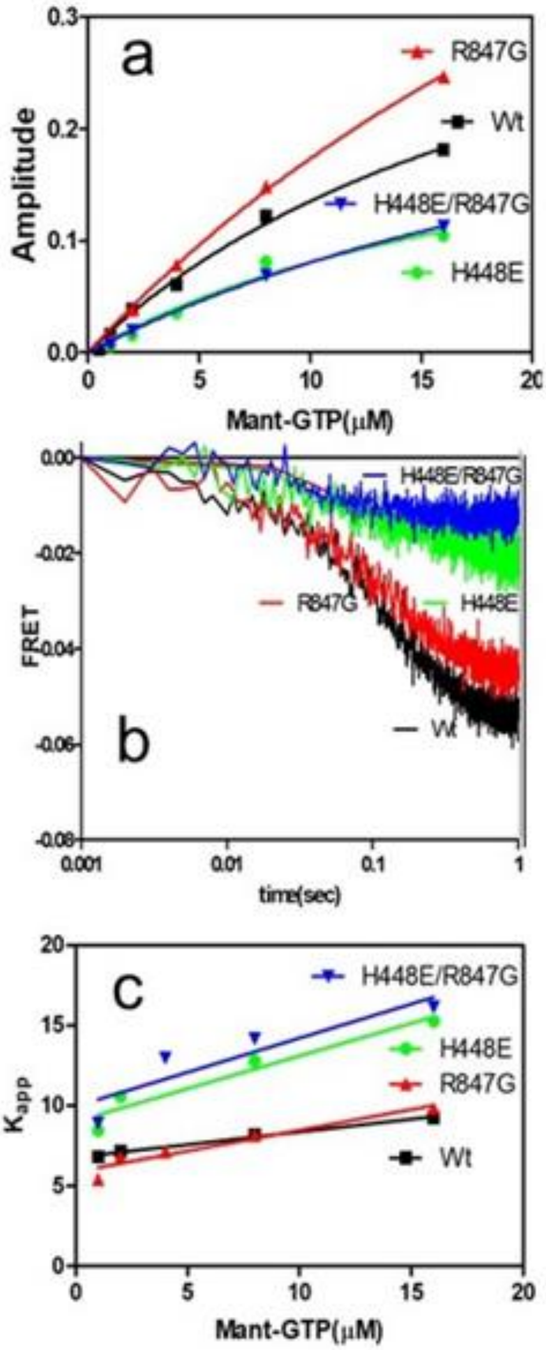

Figure S2 Interaction of Mant-GTP with IF2 wt (black), IF2 H448E (green), IF2 R847G (red) and IF2 H448E/R847G (blue) mutants. **a**) fluorescence amplitude changes upon binding Mant-GTP to IF2 molecules as a function of increasing concentrations of the nucleotide triphosphate; **b**) stopped-flow kinetic analysis of Mant-GTP dissociation from IF2 molecules monitored by the decrease of the FRET fluorescence emission using as observable the FRET signal generated by exciting the intrinsic Trp residue of IF2; **c**) variation of the K_{app} of Mant-GTP binding to IF2 molecules as a function of increasing concentrations of the nucleotides. The experiments were carried out with a Kintek Model SF-2004 stop-flow apparatus (Figure S4).The IF2 molecules bearing the H448E substitution bind GTP faster and dissociate GTP more slowly than IF2 wt and IF2 R847G. Thus, the reduced fluorescence amplitude observed with these mutants suggests that the conformation in these mutants is somewhat altered.

a)

5'GGGAAUUCAAAAAUUUAAAAGUUAAC**<u>AGGU</u>**AUACAUACU**<u>AUG</u>**UUUACGAU
UACUACGAUCUUCUUCACU**<u>UAA</u>**CGCGUCUGCAGGCAUGCAAGCU-3'.

b)

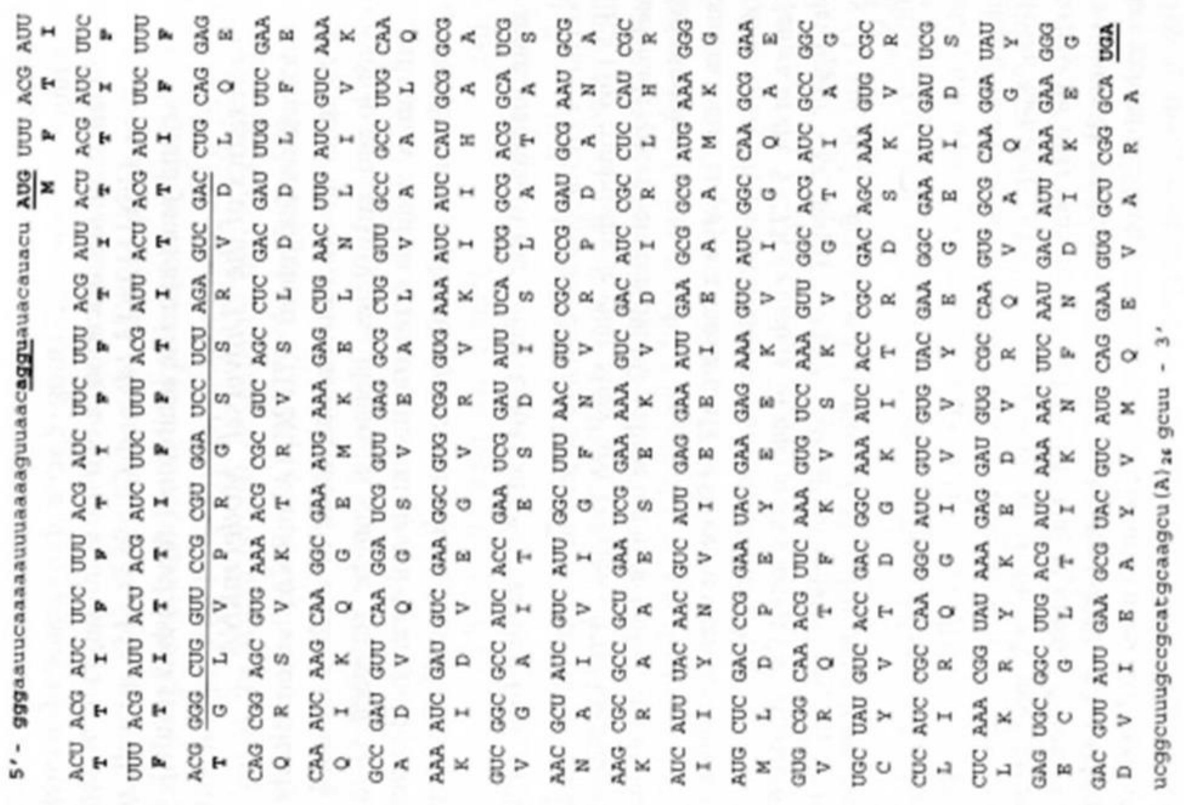

Figure S3 Sequence of the mRNAs used in the translation tests with IF2 wt and mutants. The Shine-Dalgarno sequences, the initiation and termination triplets are written in bold
a)Sequence of 022mRNA:
5'GGGAAUUCAAAAAUUUAAAAGUUAAC**<u>AGGU</u>**AUACAUACU**<u>AUG</u>**UUUACGAUUACUACGAUCUUCUUCACU**<u>UAA</u>**CGCGUCUGCAGGCAUGCAAGCU-3'. The sequence is taken from [1].
b)Sequence of 027IF2CmRNA. The Shine-Dalgarno sequence, the initiation and termination triplets are written in bold and underlined. The 5'UTR and 3'UTR are in lower case letters and the coding bases in capital letters. The amino acids of the 027 peptide (bold letters) and *B. stearothermophilus* IF2C domain (non-bold) sequences are indicated below the corresponding codons. The underlined nucleotide sequence corresponds to the spacer encoding a thrombin-sensitive peptide. From [2]

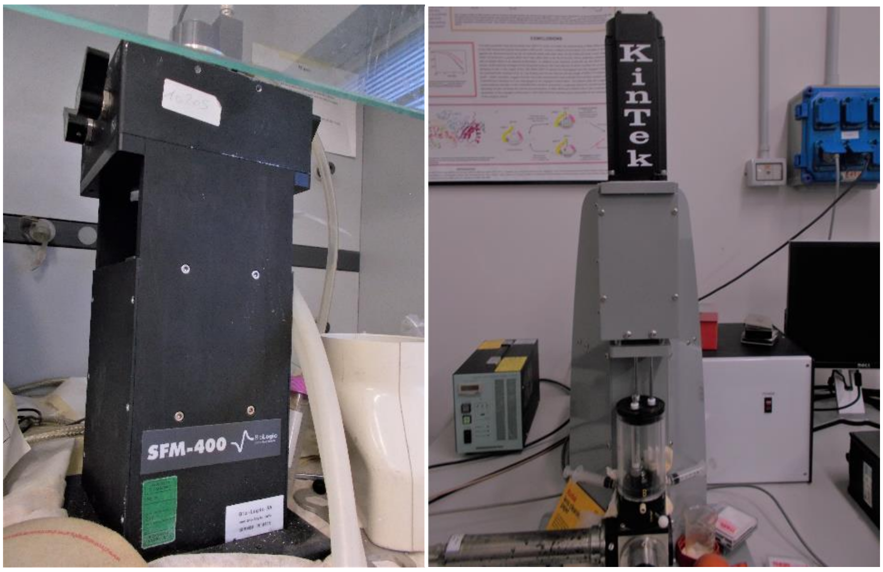

Fig. S4 Left: Biologique SFM-400 quench-flow apparatus used to analyze the kinetics of IF2-dependent GTP hydrolysis and initiation dipeptide formation in the presence of IF2 wt and IF2 mutants. Right: Kintek Model SF-2004 stop-flow apparatus used for studying the interaction of IF2 wt and IF2 mutants with Mant-GTP.

References

1 La Teana, A.; Pon, C.L.; Gualerzi, C.O. Translation of mRNAs with degenerate initiation triplet AUU displays high initiation factor 2 dependence and is subject to initiation factor 3 repression. Proc. Natl. Acad. Sci. U.S.A. 1993, 90, 4161-4165.

2 Brandi, L.; Dresios, J.; Gualerzi, C.O. Assays for the identification of inhibitors targeting specific translational steps. Meth. Mol. Med. New Antibiotic targets 2007, 142, 87-105.
